# Accuracy of using automated methods for detecting adverse events from electronic health record data: a research protocol

**DOI:** 10.1186/s13012-014-0197-6

**Published:** 2015-01-08

**Authors:** Christian M Rochefort, David L Buckeridge, Alan J Forster

**Affiliations:** Ingram School of Nursing, Faculty of Medicine, McGill University, Wilson Hall, 3506 University Street, Montreal, QC H3A 2A7 Canada; McGill Clinical and Health Informatics Research Group, McGill University, 1140, Pine Avenue West, Montreal, QC H3A 1A3 Canada; Department of Epidemiology, Biostatics and Occupational Health, Faculty of Medicine, McGill University, Purvis Hall, 1020 Pine Avenue West, Montreal, QC H3A 1A2 Canada; Ottawa Hospital Research Institute, Ottawa, ON Canada; The Ottawa Hospital, 725 Parkdale Ave, Ottawa, ON K1Y 4E9 Canada

**Keywords:** Adverse events, Electronic health record, Acute care hospital, Automated detection, Natural language processing, Patient safety, Data warehouse

## Abstract

**Background:**

Adverse events are associated with significant morbidity, mortality and cost in hospitalized patients. Measuring adverse events is necessary for quality improvement, but current detection methods are inaccurate, untimely and expensive. The advent of electronic health records and the development of automated methods for encoding and classifying electronic narrative data, such as natural language processing, offer an opportunity to identify potentially better methods. The objective of this study is to determine the accuracy of using automated methods for detecting three highly prevalent adverse events: a) hospital-acquired pneumonia, b) catheter-associated bloodstream infections, and c) in-hospital falls.

**Methods/design:**

This validation study will be conducted at two large Canadian academic health centres: the McGill University Health Centre (MUHC) and The Ottawa Hospital (TOH). The study population consists of all medical, surgical and intensive care unit patients admitted to these centres between 2008 and 2014. An automated detection algorithm will be developed and validated for each of the three adverse events using electronic data extracted from multiple clinical databases. A random sample of MUHC patients will be used to develop the automated detection algorithms (cohort 1, development set). The accuracy of these algorithms will be assessed using chart review as the reference standard. Then, receiver operating characteristic curves will be used to identify optimal cut points for each of the data sources. Multivariate logistic regression and the areas under curve (AUC) will be used to identify the optimal combination of data sources that maximize the accuracy of adverse event detection. The most accurate algorithms will then be validated on a second random sample of MUHC patients (cohort 1, validation set), and accuracy will be measured using chart review as the reference standard. The most accurate algorithms validated at the MUHC will then be applied to TOH data (cohort 2), and their accuracy will be assessed using a reference standard assessment of the medical chart.

**Discussion:**

There is a need for more accurate, timely and efficient measures of adverse events in acute care hospitals. This is a critical requirement for evaluating the effectiveness of preventive interventions and for tracking progress in patient safety through time.

## Background

Adverse events (AEs) are injuries caused by the medical management rather than the underlying condition of the patient [1]. AEs are estimated to occur in 2.9%–16.6% of all acute care hospitalizations, and studies have suggested that 30%–58% of all AEs are preventable [2-9]. Preventable AEs are a leading cause of death, resulting in 44,000–98,000 deaths in acute care hospitals each year [1,3,5,6]. Preventable AEs also represent a sizeable cost to our fragile economy, with total annual cost being estimated to range between USD $17 and $29 billion due to lost income, lost household production, disability, and health care costs [4]. For these reasons, preventing AEs has been identified as a high priority worldwide [10,11]. An important requirement to evaluate the success of preventive measures is to have access to accurate, timely and efficient methods for monitoring AE rates. However, at present, there are no such methods.

Indeed, while manual chart review is the reference standard in many AE detection studies, it is a time-consuming, resource-intensive and costly process. As a consequence, it is an impractical means for the routine detection and monitoring of AEs [12-14]. As for prevalence surveys, they are very costly to implement. For this reason, they are usually limited to small cross-sectional samples of at-risk patients or to selected hospital units. Regarding incident and accident reports, studies have found that compared to manual chart review they underestimate the true incidence of AEs by a factor of about 20 [15,16]. Lastly, while discharge diagnostic codes have the advantage of being readily available, relatively inexpensive, and easy to use, [14,15] prior studies have found that they generally have low to moderate sensitivity and positive predictive value for identifying AEs [17-19]. In addition, in many jurisdictions, discharge diagnostic codes are not dated with precision. As a consequence, it can be difficult to determine whether a given code (e.g. pulmonary embolism) represents an event that occurred before the patient was hospitalized (i.e. a comorbid condition) or during the actual hospitalization (i.e. an AE) [20,21]. Moreover, discharge diagnostic codes usually become available several months after discharge, which makes them impractical for timely AE detection and monitoring. In summary, the limitations in existing methods for measuring AEs have not only curtailed the ability to conduct continuous quality monitoring in acute care hospitals but also to pursue important investigational work on potentially preventable causes of AEs. To move the field forward, there is a need for more accurate, timely and efficient methods of AE detection.

With the advent of electronic health records (EHR) and digital capture of progress notes and other clinical data—which were paralleled with the development of automated methods for encoding and classifying electronic clinical narratives—an exciting opportunity has emerged to identify potentially better methods of AE detection. Taking advantage of this new opportunity, the purpose of this study is to determine the accuracy of using automated algorithms for detecting AEs from EHR data.

### Literature review

#### Pilot work: novel methods of AE detection

With the increasing availability of electronic clinical data, preliminary investigations have attempted to use a variety of electronic triggers (e.g. an abnormal laboratory test result such as an elevated white blood cell count or the prescription of an antidote drug) as a means to identify potential cases of AEs. While these methods offer promise for detecting AEs, the reported sensitivities and positive predictive values are low, suggesting that many AEs may go undetected, and that many of the events that are identified are indeed false positives [13].

More recently, researchers have started to use alternative and potentially more accurate approaches to AE detection, such as natural language processing (NLP) of dictated electronic radiology reports or discharge summaries [22,23]. NLP refers to automated methods for converting free-text data into computer-understandable format [24]. NLP techniques have been divided into two broad categories: symbolic and statistical. Symbolic NLP techniques use the characteristics of the language (i.e. semantics, syntax and the relationships among sentences) to interpret a narrative document to the extent necessary for encoding it into one of a set of predefined categories (e.g. positive or negative for pneumonia) [25,26]. As for statistical NLP techniques, they use the frequency distribution of words and phrases to automatically classify a set of narrative documents [27,28].

The results of preliminary studies that have implemented these NLP techniques are encouraging [29,30]. For instance, we recently conducted a pilot study to validate the accuracy of using statistical NLP for identifying cases of deep vein thrombosis (DVT) and pulmonary embolism (PE) from free-text electronic narrative radiology reports [31]. This method was found to be highly effective and accurate. The statistical NLP model predicting DVT achieved sensitivity of 0.80 (95% CI: 0.76–0.85), specificity of 0.98 (98% CI: 0.97–0.99) and positive predictive value (PPV) of 0.89 (95% CI: 0.85–0.93). As for the statistical NLP model predicting PE, sensitivity was 0.79 (95% CI: 0.73–0.85), specificity 0.99 (95% CI: 0.98–0.99), and PPV was 0.84 (95% CI: 0.75–0.92) [31].

However, narrative data alone may not contain all the information needed to accurately identify all types of AEs. For some events, such as hospital-acquired pneumonia (HAP), it might be necessary to combine the information from a variety of electronic data sources, including narrative chest x-ray reports, microbiology and laboratory data, and progress notes to accurately identify positive cases [13]. To move this field forward, as well as to maximize the accuracy of AE detection, there is a need for comprehensive automated AE detection algorithms that integrate the information from all the available data sources (e.g. microbiology and laboratory results, free-text radiology reports and progress notes and electronic vital signs). The primary objective of this study, and the original contribution of this research proposal, is to assess the accuracy of using comprehensive automated algorithms for detecting AEs in acute care hospitals. A secondary objective of this study is to determine the external validity of these algorithms.

## Methods

### Settings

This study will be conducted at two leading Canadian academic health centres: the McGill University Health Centre (MUHC) and The Ottawa Hospital (TOH). The MUHC is composed of five acute care hospitals and has more than 800 adult beds. It serves a population of 1.7 million people (22% of Quebec’s population), with an annual volume of 735,000 ambulatory visits, 33,300 surgeries, and 40,000 hospitalizations [32]. TOH is composed of three campuses serving a community of more than one million people across the National Capital Region and Eastern Ontario. It is composed of 1,149 beds, with annual volumes of more than 1,000,000 ambulatory care visits, 34,000 surgeries, and 48,000 patient admissions [33]. TOH was the first academic health centre in Canada to implement a research data warehouse as part of its clinical information system and to pioneer AE detection and monitoring through new information technologies and the secondary use of electronic clinical data.

### Design and population

The study population will consist of all adult medical, surgical and intensive care unit patients admitted to the MUHC and TOH between January 1, 2008 and December 31, 2014. First, a random sample of MUHC patients will be used to develop baseline automated AE detection algorithms (cohort 1, development set). These baseline algorithms will be developed using published definitions (e.g. National Healthcare Safety Network/Centers for Disease Control and Prevention (CDC/NHSN) definitions of nosocomial infections), which will also guide the extraction of the required data from the various electronic clinical databases at the MUHC and TOH. Then, the accuracy of these baseline algorithms will be assessed in comparison to information contained in the medical charts. To maximize the accuracy of AE detection, these algorithms will then be iteratively optimized by varying the cutoff values for each of the data sources, as well as the type and the number of data sources included in the algorithms. The most accurate algorithms will then be validated on a distinct random sample of MUHC patients (cohort 1, validation set). To determine the robustness of these algorithms and the extent to which they can be generalized to other acute care settings, the most accurate algorithms developed and validated at the MUHC will be applied to a random sample of TOH patients (cohort 2), and a reference standard assessment of the medical chart will be performed.

### Data sources and data extraction

Data required to develop the automated algorithms will be extracted from the MUHC and TOH clinical data warehouses and will be linked by unit, patient and hospital admission date and time. Specifically, data will be extracted from nine electronic databases, including: 1) laboratory, 2) microbiology, 3) radiology, 4) vital signs, 5) intensive care unit, 6) pharmacy, 7) admission, discharge and transfer, 8) discharge abstracts, and 9) narrative progress notes.

### Approach

#### Development of automated AE detection algorithms

For the purpose of this study, three potentially preventable AEs were selected: a) hospital-acquired pneumonias (HAPs), including ventilator-associated pneumonias (VAPs); b) central venous catheter-associated blood stream infections (CVC-BSIs); and c) in-hospital falls. These AEs were selected because they can each result in increased morbidity, mortality and length of hospital stay, as well as increased health care expenditures [34-42]. Moreover, these indicators have high incidence rates compared to other AEs. HAPs represent one of the most common nosocomial infections, accounting for 15% of all hospital-acquired infections and 25% of all ICU-acquired infections [34-37]. HAPs are estimated to occur at a rate of between five and ten cases per 1,000 hospital admission, and in about 8%–28% of patients receiving mechanical ventilation [34,37,43]. Central venous catheters are the single most important cause of health-care-associated bloodstream infections, with CVC-BSIs estimated to occur in 2%–6% of all catheterizations [38,39,44]. Between 3% and 27% of patients will fall at least once during their hospitalization [40,45,46].

Data from the development set (cohort 1) will be used to develop three baseline automated AE detection algorithms, one for each of the selected AEs. These baseline algorithms will be developed by applying the CDC/NHSN definitions for HAP/VAP [47] and CVC-BSI [42] and the WHO definition for a fall [48] (Table 1). A positive case of AE will be defined as any patient whose electronic data satisfies the CDC/NHSN definitions for HAP/VAP and CVC-BSI or the WHO definition for a fall (Table 1). Negative cases will be those patients that are negative on one or more of these criteria.

**Table 1:** **Data sources and criteria for determining adverse event (AE) occurrence**

**Adverse event**	**Database**	**Method for detecting**	**Criteria assessed >48 h after hospital admission (HAP, CVC-BSI) or initiation of mechanical ventilation (VAP)**
HAP/VAP	Radiology	NLP	A chest radiograph that is suggestive of a) a new, progressive or persistent infiltrate; b) consolidation; or c) cavitation
	Microbiology	Query/rule	Qualitative and quantitative reports of sputum cultures suggestive of the presence of HAP or VAP (e.g. Gram stain of respiratory secretions sample with ≥25 neutrophils and ≤10 squamous epithelial cells per high power field)
	Laboratory	Query/rule	Evidence of leukopenia (WBC < 4,000/mm^3^), leukocytosis (WBC > 12,000/mm^3^) or abnormal trends in the WBC
	Vital signs	Query/rule	Patient body temperature >38°C or abnormal trends in elevated body temperatures; Worsening gas exchanges (e.g. O_2_ desaturations, increased oxygen requirements, increased ventilator demand [⬆FiO_2_ of ≥ 0.2 point or⬆PEEP values of ≥ 3cmH_2_O compared to previous 48 h and sustained for 48 h)
	Progress notes	NLP	Evidence of altered mental status in patients ≥70 years old
	Pharmacy	Query/rule	Prescription of a new antimicrobial agent covering the micro-organisms most commonly causing HAP/VAP, duration and timing of antibiotic exposure [49].
CVC-BSI	Microbiology	Query/rule	Blood cultures that are suggestive of the presence of a CVC-BSI. To exclude cases of secondary BSI, special queries will be constructed to determine if organisms recovered from blood cultures were also recovered from non-blood cultures (e.g. sputum, surgical site or urine cultures). CDC/NHSN rules will be followed to exclude cases of positive blood cultures due to contamination from common skin commensals
	Laboratory	Query/rule	Evidence of leucopenia (WBC < 4,000/mm^3^), leukocytosis (WBC > 11,000/mm^3^) or abnormal trends in the WBC
	Vital signs	Query/rule	Body temperature >38°C or abnormal trends in elevated body temperatures >48 h after hospital admission
	Pharmacy	Query/rule	Prescription of an antibiotic covering the micro-organisms most commonly causing CVC-BSIs, duration and timing of antibiotic exposure [49]
	Radiology	NLP	To define the denominator, the radiology database will be consulted to identify chest radiograph reports showing the presence of a CVC [50]
	Progress notes	NLP	Progress notes suggestive of the presence of a CVC-BSI
In-hospital fall	Progress notes	NLP	Progress notes suggestive of an in-hospital fall (e.g. ‘patient found on the floor’, ‘patient fell off the bed’)
	Radiology	NLP	Narrative radiology reports that are suggestive of the occurrence of a fall (e.g. history of fall, s/p fall, syncope). Then, these reports will be scanned for evidence of soft tissue injuries, long bones, hip, wrist or skull fractures or traumatic brain injuries

*Abbreviations*: *NLP* Natural Language Processing, *WBC* white blood cell count, *HAP* hospital-acquired pneumonia, *VAP* ventilator-associated pneumonia, *CVC-BSI* central venous catheter-associated blood stream infections.

### Reference standard assessment of the presence of an AE

The accuracy of the baseline algorithms will be assessed using a reference standard assessment of the medical chart. Charts will be reviewed by trained medical chart reviewers (MCRs) that will be blind to the patients’ AE status (i.e. positive vs. negative) as determined by the automated algorithms. MCRs will use a standardized computer-based abstraction form that was developed during our pilot work [31]. To assess inter-rater reliability, a random sample of 10% of the medical charts will be blindly reviewed by a second MCR, and interclass correlation coefficients (ICC) will be computed. To ensure data quality throughout the study, MCRs will undergo periodic quality assurance monitoring.

### Optimization of the automated AE detection algorithms

Prior studies have shown that the CDC/NNSH definitions have limited accuracy for identifying cases of AEs [51-53]. To maximize the accuracy of AE detection, the baseline algorithms for HAP/VAP and CVC-BSI will be optimized using four successive steps. Steps 1–3 will use the patient medical chart as the reference standard, whereas step 4 will estimate the reference standard using latent class analyses.

*Step 1*: Receiver operating characteristic (ROC) curves will be used to identify: a) optimal cut points for defining the presence of an AE (e.g. using various threshold values for defining an elevated white blood cell count, an abnormal ventilator setting or an elevated body temperature) and b) optimal time window for measuring these parameters (e.g. requiring a single day with an elevated WBC vs. at least two consecutive days). In addition, the ROC area under curve (AUC), along with its 95%CIs, will be used to assess the accuracy of each data source.

*Step 2*: Three separate multivariate logistic regression analyses—one for each AE—will be conducted to assess the incremental effect in detection accuracy of combining data sources, using the optimal cut points and measurement windows defined in step 1. Stepwise and backward procedures will be used to identify data sources that are significantly associated with the occurrence of an AE. AUCs along with their 95%CIs will be used to assess the incremental effect in detection accuracy associated with the inclusion of a given data source in the regression model. AUCs across models will be compared using the approach described by Hanley and McNeill [54]. Data sources not significantly associated with AE occurrence will be eliminated from the model.

*Step 3*: The regression models developed in step 2 will be used to assess the incremental effect in detection accuracy of including patient demographic characteristics and comorbidities. Indeed, prior studies have found that older patients with more comorbidities are at higher risk of experiencing AEs [12]. Patient age, sex, and admission urgency (elective vs. urgent) and admission type (medical vs. surgical) will be obtained from the discharge abstract database. Comorbidities will be measured using the Charlson Comorbidity Index, a weighted index of 17 comorbidities that are associated with an increased risk of mortality [55]. Comorbidities will be extracted from discharge summaries and progress notes using a previously validated NLP system described by Chuang et al. [56]. Then, patient age and comorbidities will be entered as continuous variables in the regression models, whereas patient sex and admission urgency and type will be included as dichotomous variables. The AUCs of models containing demographic characteristics and comorbidities will be compared to that of models not containing these characteristics [54].

*Step 4*: The limitations associated with using the patient medical charts as the reference standard for determining the true AE status (e.g. omissions, errors) [13] will be acknowledged as these limitations may influence the perceived accuracy of the automated AE detection algorithms in either direction [57]. As such, the analyses from steps 1–3 will be repeated using latent class analysis to estimate the reference standard. Latent class analyses will be conducted assuming the conditional dependence of the data sources [57]. The AUCs of the best performing algorithms from step 1 to step 3 will then be compared to that of the best performing models from step 4 [54].

Lastly, the best performing algorithms from the development steps will be applied to the validation set (cohort 1) and their performance will be assessed using the AUCs. AUCs from the validation set will then be compared to those obtained during the development steps [54]. In each of the development steps (steps 1–4) and validation steps estimates of sensitivity, specificity, positive and negative predictive value, along with their 95% confidence intervals, will be computed for the best performing algorithms.

### External validation of the automated AE detection algorithms

To assess the robustness and the extent to which the best performing algorithms developed and validated at the MUHC can be generalized to other acute care hospitals, they will be applied to a second cohort (cohort 2) consisting of medical, surgical and intensive care unit patients admitted to TOH. A reference standard assessment of the medical chart will then be performed, and the AUCs obtained from cohort 2 will be compared to those obtained from the best performing algorithms in cohort 1 to determine if there are significant differences in the performance of the algorithms across sites.

### Sample size requirements

To maximize efficiency, AE positive patients will be oversampled in relation to AE negative patients [58]. To avoid bias due to seasonal trends, [59,60] each AE positive patient will be matched with AE negative patients who will be sampled among those that were admitted on the same date. In addition, to minimize the costs associated with performing chart review, all AE negative patients will be selected so that they are negative for all three AEs according to the automated AE detection algorithms.

Assuming an incidence rate of 5.0% for both HAP/VAP [34,37,43] and CVC-BSIs [38,39,44] and of 7.0% for in-hospital falls, [40,45] a total of 639 AE positive charts and 3,099 AE negative charts will be reviewed at the MUHC, using the worst case scenario (see Tables 2 and 3), to generate a 95% confidence interval width of 0.10 around a sensitivity estimate of 0.90 that is adjusted for the over-sampling of AE positive patients [58]. Similar figures will be required at TOH to assess the external validity of the automated AE algorithms.

**Table 2:** **Sample sizes** ^**a**^ **required for a 95% confidence interval width of 0.10 around the sensitivity estimate adjusted for the over-sampling of adverse event (AE) positive patients induced by the study design**

**Adverse event (AE) indicator**	**Estimated incidence rate** ^**b**^	**Desired specificity**	**Expected corrected sensitivity (range)**	**Expected positive predictive value (range)**	**Optimal sampling test positivity rate**	**Optimal sample sizes** ^**c**^		
						**No. of AE positive patients**	**No. of AE negative patients**	**Total no. of patients sampled**
Nosocomial pneumonia (NP)	0.05	0.95	0.85	0.80	0.10290	191	1,980	2,171
				0.90	0.10290	94	979	1,073
			0.90	0.80	0.07104	*237*	*3,099*	*3,336*
				0.90	0.07104	117	1,532	1,649
		0.98	0.85	0.80	0.06667	119	1,975	2,094
				0.90	0.06667	59	976	1,035
			0.90	0.80	0.04545	148	3,111	3,259
				0.90	0.04545	73	1,538	1,611
Central venous catheter-associated blood stream infection (CVC-BSI)	0.05	0.95	0.85	0.80	0.10290	191	1,980	2,171
				0.90	0.10290	94	979	1,073
			0.90	0.80	0.07104	*237*	*3*,*099*	*3,336*
				0.90	0.07104	117	1,532	1,649
		0.98	0.85	0.80	0.06667	119	1,975	2,094
				0.90	0.06667	59	976	1,035
			0.90	0.80	0.04545	148	3,111	3,259
				0.90	0.04545	73	1,538	1,611
In-hospital fall	0.07	0.95	0.85	0.80	0.10290	133	1,379	1,512
				0.90	0.10290	66	682	747
			0.90	0.80	0.07104	*165*	*2,161*	*2,327*
				0.90	0.07104	82	1,068	1,150
		0.98	0.85	0.80	0.06667	83	1,376	1,458
				0.90	0.06667	41	680	721
			0.90	0.80	0.04545	103	2,170	2,273
				0.90	0.04545	51	1,073	1,124

^a^Sample size calculations are based on [58]. ^b^Based on the literature and expert opinion. ^c^Values in italics represent the worst case scenario for a given AE indicator.

**Table 3:** **Calculation for establishing the required sample size, assuming the worst case scenario for each adverse event (AE) indicator**

**Adverse event (AE) indicator**	**Worst case scenario** ^**a**^	
	**AE positive**	**AE negative** ^**b**^
Nosocomial pneumonia (NP)	237	3,099
Central venous catheter-associated blood stream infection (CVC-BSI)	237	3,099
In-hospital fall	165	2,161
Total	639	3,099
Total number of charts to be reviewed if worst case scenario	3,738	

^a^Based on optimal sample sizes provided in Table 2 (see values in italics). ^b^To minimize the costs associated with performing chart review, all AE negative patients will be selected so that they are negative for all three AEs according to the automated detection algorithms. As such, 3,099 is the largest number of AE negative patients required, assuming the worst case scenario.

## Discussion

### Current study status

A considerable amount of time and effort were required during the first 2 years of the study for extracting, cleaning and formatting the required data, as well as for meeting confidentiality and security requirements for data extraction and use at each site. At present time, we have developed and validated a statistical NLP model for identifying narrative radiology reports that are suggestive of the presence of pneumonia. We are now in the process of developing the pneumonia detection algorithm using this NLP-based information along with data from other electronic sources (e.g. microbiology and laboratory results, electronic vital signs). The development of the CVC-BSI algorithm is also underway.

### Relevance and impact

This study will likely produce more accurate and timely measures of AEs. Three important contributions of this study should be emphasized. First, we propose to integrate several sources of EHR data into comprehensive automated AE detection algorithms. This approach contrasts with previous algorithms, [29,30,61-66] which were for the most part based on a single source of EHR data (e.g. electronic triggers or narrative radiology reports). Second, we will attempt to maximize accuracy by optimizing the automated algorithms, an approach that also departs from prior studies. Lastly, we will determine if the automated AE detection algorithms developed in one setting can be generalized to other similar clinical contexts; a question that has rarely been addressed in prior investigations.

The measures that will be developed and validated in this study are likely to have multiple applications. Hospitals could use these measures to assess the incidence rates of AEs, develop near real-time monitoring systems, evaluate different interventions aimed at improving patient safety, track progress in patient safety through time, or benchmark performance at the local or national levels. In addition, because these methods are automated, they offer the potential to rapidly scan large numbers of patient records and EHR data with minimal human effort, a major gain compared to using manual chart review. Moreover, because these methods will be applied to electronic data that have the advantage of being time and date stamped (e.g. radiology reports), they can potentially determine the timing of AE occurrence with relatively high precision; a net improvement compared to using discharge diagnostic codes and a key requirement for determining how antecedent exposures are related to the risk of AE.

## References

[1] Kohn LT, Corrigan J, Donaldson MS (2000). To Err is Human. Building a Safer Health System.

[2] Brennan TA, Leape LL, Laird NM, Hebert L, Localio AR, Lawthers AG (1991). Incidence of adverse events and negligence in hospitalized patients. Results of the Harvard Medical Practice Study I. N Engl J Med.

[3] Leape LL, Brennan TA, Laird N, Lawthers AG, Localio AR, Barnes BA (1991). The nature of adverse events in hospitalized patients. Results of the Harvard Medical Practice Study II. N Engl J Med.

[4] Thomas EJ, Studdert DM, Burstin HR, Orav EJ, Zeena T, Williams EJ (2000). Incidence and types of adverse events and negligent care in Utah and Colorado. Med Care.

[5] Thomas EJ, Studdert DM, Newhouse JP, Zbar BI, Howard KM, Williams EJ (1999). Costs of medical injuries in Utah and Colorado. Inquiry.

[6] Baker GR, Norton PG, Flintoft V, Blais R, Brown A, Cox J (2004). The Canadian Adverse Events Study: the incidence of adverse events among hospital patients in Canada. CMAJ.

[7] Davis P, Lay-Yee R, Briant R, Ali W, Scott A, Schug S. Adverse events in New Zealand public hospitals I: occurrence and impact. N Z Med J. 2002;115(1167):U271. 12552260

[8] Davis P, Lay-Yee R, Briant R, Ali W, Scott A, Schug S. Adverse events in New Zealand public hospitals II: preventability and clinical context. N Z Med J. 2003;116(1183):U624. 14581938

[9] Wilson RM, Runciman WB, Gibberd RW, Harrison BT, Newby L, Hamilton JD (1995). The Quality in Australian Health Care Study. Med J Aust.

[10] The Research Priority Setting Working Group (2008). Global Priorities for Research in Patient Safety.

[11] Aspden P, Corrigan JM, Wolcott J, Erickson SM (2004). Patient Safety: Achieving a New Standard for Care.

[12] Govindan M, Van Citters AD, Nelson EC, Kelly-Cummings J, Suresh G. Automated detection of harm in healthcare with information technology: a systematic review. Qual Saf Health Care. 2010;19(5):e11. 10.1136/qshc.2009.033027 20671081

[13] Murff HJ, Patel VL, Hripcsak G, Bates DW (2003). Detecting adverse events for patient safety research: a review of current methodologies. J Biomed Inform.

[14] Klompas M, Yokoe DS (2009). Automated surveillance of health care-associated infections. Clin Infect Dis.

[15] Bates DW, Evans RS, Murff H, Stetson PD, Pizziferri L, Hripcsak G (2003). Detecting adverse events using information technology. J Am Med Inform Assoc.

[16] Classen DC, Resar R, Griffin F, Federico F, Frankel T, Kimmel N (2011). 'Global trigger tool' shows that adverse events in hospitals may be ten times greater than previously measured. Health Aff (Millwood).

[17] Kaafarani HM, Borzecki AM, Itani KM, Loveland S, Mull HJ, Hickson K (2011). Validity of selected patient safety indicators: opportunities and concerns. J Am Coll Surg.

[18] Goto M, Ohl ME, Schweizer ML, Perencevich EN (2014). Accuracy of administrative code data for the surveillance of healthcare-associated infections: a systematic review and meta-analysis. Clin Infect Dis.

[19] Romano PS, Mull HJ, Rivard PE, Zhao S, Henderson WG, Loveland S (2009). Validity of selected AHRQ patient safety indicators based on VA National Surgical Quality Improvement Program data. Health Serv Res.

[20] Houchens RL, Elixhauser A, Romano PS (2008). How often are potential patient safety events present on admission?. Jt Comm J Qual Patient Saf.

[21] Bahl V, Thompson MA, Kau TY, Hu HM, Campbell DA (2008). Do the AHRQ patient safety indicators flag conditions that are present at the time of hospital admission?. Med Care.

[22] Murff HJ, Forster AJ, Peterson JF, Fiskio JM, Heiman HL, Bates DW (2003). Electronically screening discharge summaries for adverse medical events. J Am Med Inform Assoc.

[23] Forster AJ, Andrade J, van Walraven C (2005). Validation of a discharge summary term search method to detect adverse events. J Am Med Inform Assoc.

[24] Allen J (1995). Natural Language Understanding.

[25] Nadkarni PM, Ohno-Machado L, Chapman WW (2011). Natural language processing: an introduction. J Am Med Inform Assoc.

[26] Chapman WW, Wagner MM, Moore AW, Aryel RM (2006). Natural Language Processing for Biosurveillance. Handbook of Biosurveillance.

[27] Mitchell TM (1997). Machine Learning.

[28] Hastie T, Tibshirani R, Friedman J (2009). The Elements of Statistical Learning. Data mining, Inference, and Prediction. Secondth edition.

[29] Murff HJ, FitzHenry F, Matheny ME, Gentry N, Kotter KL, Crimin K (2011). Automated identification of postoperative complications within an electronic medical record using natural language processing. JAMA.

[30] FitzHenry F, Murff HJ, Matheny ME, Gentry N, Fielstein EM, Brown SH (2013). Exploring the frontier of electronic health record surveillance: the case of postoperative complications. Med Care.

[31] Rochefort CM, Verma AD, Eguale T, Lee TC, Buckeridge DL: A novel method of adverse event detection can accurately identify venous thromboembolism (VTE) from narrative electronic health record data. *JAMIA* 2014. doi:10.1136/amiajnl-2014-002768. 10.1136/amiajnl-2014-002768 PMC4433368 25332356

[32] McGill University Health Centre (MUHC): MUHC at a glance [http://muhc.ca/homepage/page/muhc-glance]

[33] The Ottawa Hospital: the Ottawa Hospital in statistics [http://www.ottawahospital.ca/wps/portal/Base/TheHospital/AboutOurHospital/Statistics]

[34] Chastre J, Fagon JY (2002). Ventilator-associated pneumonia. Am J Respir Crit Care Med.

[35] American Thoracic Society and Infection Disease Society of America (2005). Guidelines for the management of adults with hospital-acquired, ventilator-associated, and healthcare-associated pneumonia. Am J Respir Crit Care Med.

[36] Kieninger AN, Lipsett PA (2009). Hospital-acquired pneumonia: pathophysiology, diagnosis, and treatment. Surg Clin North Am.

[37] Joseph NM, Sistla S, Dutta TK, Badhe AS, Parija SC (2010). Ventilator-associated pneumonia: a review. Eur J Intern Med.

[38] Hockenhull JC, Dwan K, Boland A, Smith G, Bagust A, Dundar Y (2008). The clinical effectiveness and cost-effectiveness of central venous catheters treated with anti-infective agents in preventing bloodstream infections: a systematic review and economic evaluation. Health Technol Assess.

[39] Maki DG, Kluger DM, Crnich CJ (2006). The risk of bloodstream infection in adults with different intravascular devices: a systematic review of 200 published prospective studies. Mayo Clin Proc.

[40] Salgado RI, Lord SR, Ehrlich F, Janji N, Rahman A (2004). Predictors of falling in elderly hospital patients. Arch Gerontol Geriatr.

[41] Patel PJ, Leeper KV, McGowan JE (2002). Epidemiology and microbiology of hospital-acquired pneumonia. Semin Respir Crit Care Med.

[42] O'Grady NP, Alexander M, Burns LA, Dellinger EP, Garland J, Heard SO (2011). Guidelines for the prevention of intravascular catheter-related infections. Am J Infect Control.

[43] Rotstein C, Evans G, Born A, Grossman R, Light RB, Magder S (2008). Clinical practice guidelines for hospital-acquired pneumonia and ventilator-associated pneumonia in adults. Can J Infect Dis Med Microbiol.

[44] Eggimann P, Pittet D (2002). Overview of catheter-related infections with special emphasis on prevention based on educational programs. Clin Microbiol Infect.

[45] Oliver D, Daly F, Martin FC, McMurdo ME (2004). Risk factors and risk assessment tools for falls in hospital in-patients: a systematic review. Age Ageing.

[46] Hill AM, Hoffmann T, Hill K, Oliver D, Beer C, McPhail S (2010). Measuring falls events in acute hospitals-a comparison of three reporting methods to identify missing data in the hospital reporting system. J Am Geriatr Soc.

[47] Centers for Disease Control and Prevention (CDC): CDC/NHSN surveillance definitions for specific types of infections [http://www.cdc.gov/nhsn/pdfs/pscmanual/17pscnosinfdef_current.pdf]

[48] World Health Organization (WHO): WHO Global report on fall prevention in older age [http://www.who.int/mediacentre/factsheets/fs344/en/].

[49] Hirschhorn LR, Currier JS, Platt R (1993). Electronic surveillance of antibiotic exposure and coded discharge diagnoses as indicators of postoperative infection and other quality assurance measures. Infect Control Hosp Epidemiol.

[50] Trick WE, Chapman WW, Wisniewski MF, Peterson BJ, Solomon SL, Weinstein RA (2003). Electronic interpretation of chest radiograph reports to detect central venous catheters. Infect Control Hosp Epidemiol.

[51] Emori TG, Edwards JR, Culver DH, Sartor C, Stroud LA, Gaunt EE (1998). Accuracy of reporting nosocomial infections in intensive-care-unit patients to the National Nosocomial Infections Surveillance System: a pilot study. Infect Control Hosp Epidemiol.

[52] Tejerina E, Esteban A, Fernandez-Segoviano P, Frutos-Vivar F, Aramburu J, Ballesteros D (2010). Accuracy of clinical definitions of ventilator-associated pneumonia: comparison with autopsy findings. J Crit Care.

[53] Lin MY, Hota B, Khan YM, Woeltje KF, Borlawsky TB, Doherty JA (2010). Quality of traditional surveillance for public reporting of nosocomial bloodstream infection rates. JAMA.

[54] Hanley JA, McNeil BJ (1983). A method of comparing the areas under receiver operating characteristic curves derived from the same cases. Radiology.

[55] Charlson ME, Pompei P, Ales KL, MacKenzie CR (1987). A new method of classifying prognostic comorbidity in longitudinal studies: development and validation. J Chronic Dis.

[56] Chuang JH, Friedman C, Hripcsak G (2002). A comparison of the Charlson comorbidities derived from medical language processing and administrative data. Proc AMIA Symp.

[57] Pepe M (2004). The Statistical Evaluation of Medical Test Classification and Prediction.

[58] Irwig L, Glasziou PP, Berry G, Chock C, Mock P, Simpson JM (1994). Efficient study designs to assess the accuracy of screening tests. Am J Epidemiol.

[59] Dowell SF (2001). Seasonal variation in host susceptibility and cycles of certain infectious diseases. Emerg Infect Dis.

[60] Dowell SF, Ho MS (2004). Seasonality of infectious diseases and severe acute respiratory syndrome-what we don’t know can hurt us. Lancet Infect Dis.

[61] Dublin S, Baldwin E, Walker RL, Christensen LM, Haug PJ, Jackson ML (2013). Natural Language Processing to identify pneumonia from radiology reports. Pharmacoepidemiol Drug Saf.

[62] Elkin PL, Froehling D, Wahner-Roedler D, Trusko B, Welsh G, Ma H (2008). NLP-based identification of pneumonia cases from free-text radiological reports. AMIA Annu Symp Proc.

[63] Trick WE, Zagorski BM, Tokars JI, Vernon MO, Welbel SF, Wisniewski MF (2004). Computer algorithms to detect bloodstream infections. Emerg Infect Dis.

[64] Bellini C, Petignat C, Francioli P, Wenger A, Bille J, Klopotov A (2007). Comparison of automated strategies for surveillance of nosocomial bacteremia. Infect Control Hosp Epidemiol.

[65] Hota B, Lin M, Doherty JA, Borlawsky T, Woeltje K, Stevenson K (2010). Formulation of a model for automating infection surveillance: algorithmic detection of central-line associated bloodstream infection. J Am Med Inform Assoc.

[66] Toyabe S. Detecting inpatient falls by using natural language processing of electronic medical records. BMC Health Serv Res. 2012;12:448. 10.1186/1472-6963-12-448 PMC3519807 23217016

